# Fabrication of Self-Cleaning Superhydrophobic Surfaces with Improved Corrosion Resistance on 6061 Aluminum Alloys

**DOI:** 10.3390/mi11020159

**Published:** 2020-02-01

**Authors:** Xiaojuan Dong, Jianbing Meng, Yizhong Hu, Xiuting Wei, Xiaosheng Luan, Haian Zhou

**Affiliations:** School of Mechanical Engineering, Shandong University of Technology, Zibo 255000, China; dongxiaojuan@sdut.edu.cn (X.D.); hyz576008000@163.com (Y.H.); wxt@sdut.edu.cn (X.W.); lxs631781687@163.com (X.L.); zhouhaian@sdut.edu.cn (H.Z.)

**Keywords:** superhydrophobic, sand peening, electrochemical oxidation, aluminum alloy, self-cleaning, corrosion resistance

## Abstract

Aluminum alloys are widely used, but they are prone to contamination or damage under harsh working environments. In this paper, a self-cleaning superhydrophobic aluminum alloy surface with good corrosion resistance was successfully fabricated via the combination of sand peening and electrochemical oxidation, and it was subsequently covered with a fluoroalkylsilane (FAS) film. The surface morphology, surface wettability, and corrosion resistance were investigated using a scanning electron microscope (SEM), an optical contact angle measurement, and an electrochemical workstation. The results show that binary rough structures and an FAS film with a low surface energy on the Al alloy surfaces confer good superhydrophobicity with a water contact angle of 167.5 ± 1.1° and a sliding angle of 2.5 ± 0.7°. Meanwhile, the potentiodynamic polarization curve shows that the corrosion potential has a positively shifted trend, and the corrosion current density decreases by three orders of magnitude compared with that of the original aluminum alloy sample. In addition, the chemical stability of the as-prepared superhydrophobic surface was evaluated by dripping test using solutions with different pH values for different immersion time. It indicates that the superhydrophobic surface could provide long-term corrosion protection for aluminum alloys. Consequently, the as-prepared superhydrophobic surface has excellent contamination resistance and self-cleaning efficacy, which are important for practical applications.

## 1. Introduction

Aluminum and its alloys have many advantages, such as high specific strength, good formability, low density, high specific stiffness, excellent thermal conductivity, non-magnetic properties, and so on [1,2,3,4]. They are widely used in the manufacturing fields of medical devices, as well as the aerospace and automobile industries, among others. However, the poor corrosion resistance of aluminum alloy, especially the inter-granular corrosion and pitting caused by intermetallic compound particles, greatly limits its further application [5,6]. Therefore, it is necessary to improve corrosion resistance and surface properties of aluminum alloys by surface modification. In recent years, as an essential surface treatment technology of aluminum alloy, superhydrophobic surfaces (SHS) attracted extensive attention. Darmanin and Guittard presented a very recent overview of potential applications of superhydrophobic materials [7]. They found that at least two parameters seem to be essential for many applications: the presence of air on superhydrophobic materials with self-cleaning properties and the robustness of the superhydrophobic properties. Zhang et al. introduced the fundamental theories behind superhydrophobicity followed by a comprehensive review of the recent progresses of this rapidly growing field over the past five years [8]. They found that SHS can minimize the interaction between metal substrates and aqueous corrosive species, thereby producing superior anticorrosive performance. Yu et al. reviewed the recent developments in SHS design, synthesis, and transparent manufacturing [9]. They believed that the SHS must be easily fabricated with low energy consumption and must be durable during service to extend its working life. Zhu et al. summarized the recent developments in both natural SHS and artificial SHS with various adhesions [10]. They thought that an SHS with highly controllable adhesion is feasible in different environments.

SHS refers to a surface with a water contact angle (CA) greater than 150° and a sliding angle (SA) lower than 10° [11]. Many creatures in nature, including the lotus leaf, water-strider legs, and rice leaf, show good superhydrophobic properties [12,13,14]. As we all know, the formation of SHS mainly depends on the rough morphology and chemical composition. Generally, the CA of a water droplet on a smooth surface with an extremely low surface energy can improve to 120°. Instead, the CA can reach or even exceed 150° by modifying the rough structures with low-surface-energy materials. Therefore, the key to preparing an SHS is the formation of a rough structure and a reduction in surface energy [15]. A large number of approaches were successfully used to develop superhydrophobic surfaces, including chemical etching [16], solution immersion [17], and laser machining [18]. Feng et al. reported a facile method of preparing a hierarchically structured superhydrophobic aluminum alloy surface through a one-step immersion process. The superhydrophobic aluminum alloys showed excellent anti-icing, anti-frosting, and self-cleaning properties [19]. Choi et al. presented a simple process for fabricating hierarchical aluminum surfaces with different morphologies using three different kinds of alkaline-based chemical solutions. The etched aluminum surface showed non-wetting properties, exhibiting a static CA over 150° and a dynamic CA lower than 5° for deionized water [20]. Ngo and Chun fabricated grid patterns on aluminum using a nanosecond pulse laser. After heat treatment, the as-prepared surface became superhydrophobic with a CA of 176° and an SA of 6 ± 1° [21]. However, few studies explored the corrosion resistance of SHS. 

The corrosion resistance of SHS is due to the fact that the air pockets at the solid–liquid interface can greatly inhibit the penetration of chloride ions and other corrosive substances dissolved in water into the metal matrix. Currently, the corrosion resistance of SHS can be enhanced by electrophoretic deposition, anodization, micro-arc oxidation (MAO), and so on. Zhang et al. developed a one-step electrodeposition method to fabricate SHS on aluminum. The SHS showed a significant self-cleaning and anti-corrosion ability [22]. Liu et al. fabricated a functional SHS via a one-step methodology and anodization. The SHS had a CA of as 171.9 ± 2° and a tilting angle as low as 6.2 ± 1° [23]. Zhang et al. prepared SHS on aluminum via one-step high-field anodization. The SHS with micro/nanostructured films showed a maximum CA of 163 ± 2° and an SA of less than 2° [24]. Zheng et al. constructed a superhydrophobic coating on the aluminum surface using anodization in sulfuric acid electrolyte followed by surface modification with myristic acid. The fabricated SHS exhibited good superhydrophobicity with a CA of 155.2 ± 0.5° and an SA of 3.5 ± 1.3° [25]. Zhang and Kang created an SHS with different liquid–solid contact models via the combination of MAO, phytic acid etching, and polymer plating. Compared with the substrate, the corrosion current density of the SHS in Cassie–Baxter and Wenzel models decreased about by four and three orders of magnitude, respectively [26]. These methods were mainly aimed at the original surfaces of lab-scale aluminum alloys to form SHS with rough hierarchical structures. However, most of the reported methods are still subject to some limitations, including low efficiency and high production cost. In addition, environmental problems may result if the use of unfriendly chemicals is not managed. 

Shot peening (SP) is an effective surface treatment method to increase the fatigue strength of metallic materials [27]. A large number of small and hard particles are projected onto the workpiece at high speed. Each particle impact can cause plastic deformation of the material in its vicinity. Due to the plastic strain gradient, the underlying material opposes this surface stretching, which results in favorable compressive residual stress [28]. According to the mechanism of SP, numerous pits are produced on the shot-peened surface through the plastic indentations, thereby increasing surface roughness [29]. In fact, the rough microstructure should be a good basis for the preparation of SHS [30].

In this work, we used a combination of sand peening and electrochemical oxidation (ECO) to construct micro/nano binary structures, followed by investigated (FAS) modification. SP was introduced as pretreatment to form microscale pits. However, micro-scale structures, even after low-energy modification, cannot acquire an ideal self-cleaning SHS [31]. Electrochemical oxidation can be applied for generating nano-scale structures on the pits. After treatment of SA and ECO, a hierarchical binary structure was constructed on the aluminum alloy. Following FAS modification, an SHS with a CA of 167.5 ± 1.1° and an SA of 2.5 ± 0.7° was achieved. Moreover, we investigated the effects of processing conditions on the surface wettability, including impact pressure, particle size, nozzle diameter, impact time, electrolyte concentration, working voltage, current density, oxidation time, and so on. In addition, the self-cleaning property and corrosion resistance of the SHS were studied using potentiodynamic polarization tests.

## 2. Materials and Methods

### 2.1. Materials

In this study, 6061-T6 aluminum alloys (wt.%: 0.7 Fe, 0.4–0.8 Si, 0.15–0.4 Cu, 0.15 Mg, 0.15 Mn, 0.04–0.35 Cr, 0.25 Zn, and 0.15 Ti, with the remainder being Al) [32], supplied by China Aluminum Co. Ltd. (Beijing, China), were used as the substrate materials. Sheets with a size of 40 mm × 20 mm × 2 mm were cut via wire electrical discharge machining (WEDM). Brown alumina (Al_2_O_3_) with different sizes was obtained from Ruishi Renewable Resources Group Co. Ltd. (Luoyang, China), subsequently introduced in shot peening. In addition, all chemicals were analytical grade reagents purchased from Sinopharm Chemical Reagent Co. Ltd., Shanghai, China. Deionized water was bought from Hanhong Environmental Protection Technology Co. Ltd., Zibo, China, and it was used for the preparation of all aqueous solutions.

### 2.2. Experimental Procedure

The sample sheets were mechanically polished with 1000-, 1200-, and 1500-grade metallographic sandpapers to remove the oxide or hydroxide layer of the surfaces. The polished samples were cleaned ultrasonically in sequence to remove grease with acetone, ethanol, and deionized water for 10 min. After drying treatment, the samples were sand-peened to construct microscale pits. The experimental conditions are listed in Table 1A–D, i.e., the impact pressure, particle size, nozzle diameter, and impact time, respectively. A factorial design of the experiments was used to obtain the optimization of A, B, C and D, which could provide superior microstructures for the following electrochemical oxidation. Next, the samples were ultrasonically cleaned with ethanol and deionized water sequentially for 5 min, and then dried for further use. The prepared samples were used as the working electrode, and a Pt sheet was the counter electrode. Electrochemical oxidation was performed in the aqueous solution of sodium chloride and sodium hypochlorite (0.8 M) at room temperature with magnetic stirring at a speed of 1000 rpm and different processing conditions, as listed in Table 2. The oxidized samples were then cleaned in deionized water for 10 min and dried in a vacuum oven at 80 °C. Finally, the sample sheets were immersed in an ethanol solution of fluoroalkylsilane (FAS, C_14_H_19_F_13_O_3_Si) of 1 wt.% for 1 h and kept at 120 °C for 30 min in the oven.

### 2.3. Characterization

Surface morphologies of aluminum alloys after SP and ECO were observed using a scanning electron microscope (SEM, Sirion 200, ThermoFisher Scientific, Hillsboro, NJ, USA) at 10 kV. There are two methods to measure the contact angle: one is the shape image analysis method; the other is the weighing method. The latter is usually called the wetting balance or the penetration contact angle instrument. However, the most widely used method is the shape image analysis method. Therefore, the shape image analysis method was used in this paper. The droplet was placed on the surface of the solid sample, and the shape image of the droplet was obtained through the lens and camera. Then, the contact angle of the droplet in the image was calculated via digital image processing and some algorithms. The water contact angles were measured at room temperature using an optical contact angle measuring instrument (OCA15EC, Dataphysics, Filderstadt, Germany). The sliding angles were measured using the conventional sessile-drop method. A 5-μL deionized water droplet was dropped on the obtained surface, and the average of three measurements at different positions was considered as the final contact angle. The angle at which the water droplet started to roll off the tilted surface was defined as the water sliding angle. There was no difference between the CA measurements and self-cleaning tests. A potentiodynamic polarization curve was implemented to characterize corrosion resistance at ambient temperature on the electrochemical workstation (CHI660E, CH Instruments, Austin, TX, USA).

## 3. Results and Discussion

### 3.1. Surface Wettability

**Figure 1:**
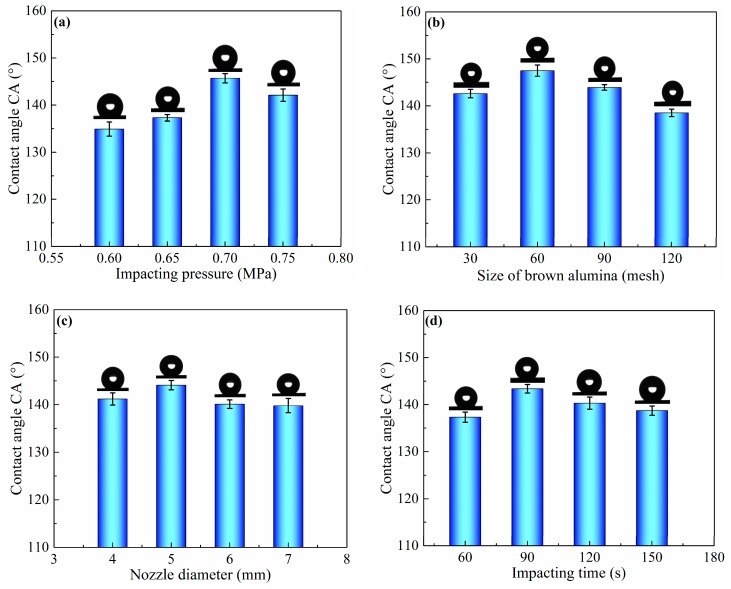
Contact angle (CA) of sample surfaces obtained by sand peening using different process parameters, such as (**a**) impacting pressure, (**b**) size of brown alumina, (**c**) nozzle diameter, and (**d**) impacting time.

To investigate the surface wettability of as-prepared aluminum alloy samples, a group of experiments were carried out, as listed in Table 1. The effect of sand peening parameters, such as impacting pressure, nozzle diameter, impacting time, and size of brown alumina, on the surface wettability was also studied using the water CA of Al alloy samples. The SP process was carried out at different impacting pressure from 0.6 to 0.75 MPa using a nozzle parameter of 5 mm and brown alumina particles of 60 mesh for 90 s. The result is shown in Figure 1a. It can be found that the CA increased from 134.9° ± 1.5° to 145.7° ± 1.0° with the increase in impacting pressure from 0.6 to 0.7 MPa, and then decreased to 142.1° ± 1.3° when the impacting pressure reached 0.75 MPa. In addition, the size of brown alumina had great influence on the CA of the sand-peened sample surface, as shown in Figure 1b. It can be observed that the CA increased with the decrease in alumina size. It reached the highest value of 147.5° ± 1.2° when the mesh of brown alumina was 60, and then decreased to 143.9° ± 0.6° and 138.5° ± 0.8° when the mesh of shot particles was 90 and 120, respectively. In addition, the effect of nozzle diameter on CA was also investigated at 0.7 MPa of impacting pressure with 60 mesh of alumina particles and 5 mm of nozzle diameter for 90 s. From Figure 1c, it can be seen that the CA increased from 141.2° ± 1.3° to 144.1° ± 1.0° with the extension of the nozzle diameter from 4 to 5 mm, and then decreased slightly to 139.8° ± 1.5° when the nozzle diameter was 7 mm. Figure 1d shows the effect of impacting time on the CA of the sample surface when the impacting pressure was 0.7 MPa, the size of brown alumina was 60 mesh, and the nozzle diameter was 5 mm. It can be found in Figure 1d that the CA gradually increased with the extended impacting time, and it reached the maximum value of 143.4° ± 0.9° after 90 s, before decreasing to 138.7° ± 1.0° when the impacting time was 150 s.

As shown in Figure 1, the CA of the sample surface increased to 147.5° ± 1.2° after sand peening and the modification of FAS, suggesting that the wettability of the as-prepared surface switched from hydrophilic to hydrophobic. However, the CA of a water droplet on the aluminum alloy sand-peened and modified with FAS was less than 150°. Consequently, the as-prepared sample surface with microscale pits could not trap enough air underneath the water droplet to make the surface superhydrophobic in comparison with the hierarchical structure. Thus, nanoscale structures were added to the as-prepared microscale pits via electrochemical oxidation. In order to provide superior microstructures for the secondary nanostructures, SP was carried out through the optimization of impacting pressure, alumina size, nozzle diameter, and impacting time via an orthogonal test, as shown in Table 1 and Table 3. The optimum parameters, including impacting pressure of 0.65 MPa, particle size of 120 mesh, nozzle diameter of 5 mm, and impacting time 150 s, were obtained using the range analysis method [33]. After sand peening with the above optimum parameters, the sample was cleaned and immersed in an aqueous solution of sodium chloride and sodium hypochlorite to construct the secondary nanostructure on the primary micro pit via electrochemical oxidation (ECO).

**Figure 2:**
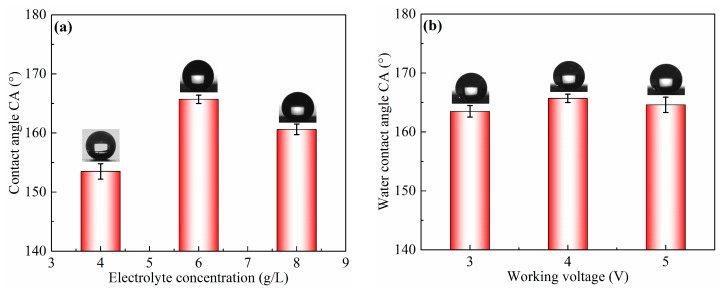

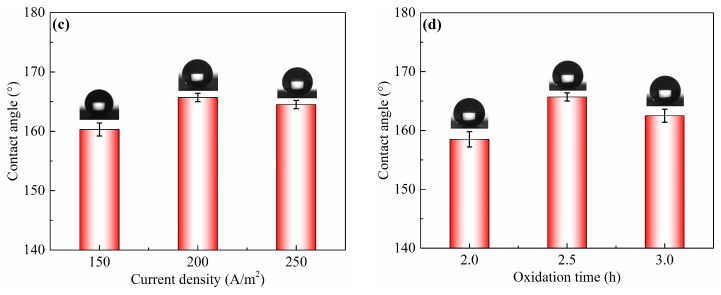
CA of sample surfaces obtained via electrochemical oxidation using different process parameters, such as (**a**) electrolyte concentration, (**b**) working voltage, (**c**) current density, and (**d**) oxidation time.

In the ECO process, electrolyte concentration, working voltage, current density, and oxidation time have important effects on the surface wettability, as shown in Figure 2. ECO was carried out at different electrolyte concentrations ranging from 4 to 8 g/L at 4 V of working voltage and 200 A/m^2^ of current density for 2.5 h, as listed in Table 2. The result is shown in Figure 2a. The CA of the as-prepared sample surface significantly increased from 153.5° ± 1.3° to 165.7° ± 0.7° with the increase in electrolyte concentration from 4 to 6 g/L, and then decreased to 160.6° ± 0.9° when the electrolyte concentration was 8 g/L. The effect of working voltage was also investigated with 6 g/L of electrolyte concentration at 200 A/m^2^ of current density for 2.5 h, and the result is shown in Figure 2b. It can be observed that CA slightly increased with the increase in working voltage. It reached the highest value of 165.7° ± 0.7° when the working voltage was 4 V, and then decreased to 164.4° ± 1.3° but remained above 150°. Figure 2c shows the change in CA on the sample surface treated at 4 V of working voltage and different current density for 2.5 h with a 6 g/L electrolyte concentration. As shown in Figure 2c, the CA evidently increased from 160.3° ± 1.1° to 165.7° ± 0.7° with the extension of current density from 150 to 200 A/m^2^. When the current density increased to 250 A/m^2^, the CA decreased to 164.5° ± 0.7°. This result shows that the better superhydrophobicity with CA of 165.7° ± 0.7° could be obtained via ECO with 200 A/m^2^ of current density. In addition, the oxidation time also had a significant influence on CA of the as-prepared sample surface. From Figure 2d, CA gradually increased with extended oxidation time, and it reached 165.7° ± 0.7° after 2.5 h, before decreasing to 162.5° ± 1.1° after 3 h, while remaining above 150°.

As shown in Figure 2, the CA of the sample surface increased from 147.5° ± 1.2° to 165.7° ± 0.7° after sand peening, electrochemical oxidation, and FAS modification. The shape of a water droplet on the as-prepared sample surface converted to an almost perfect spherical shape, with the nanostructure being fabricated on the microscale pits, due to the large amount of air trapped underneath the water droplet. Therefore, it can be concluded that surface superhydrophobicity should result from the combination of primary microstructures and second nanostructures, obtained by sand peening and electrochemical oxidation, respectively.

### 3.2. Surface Morphology and Self-Cleaning Performance

**Figure 3:**
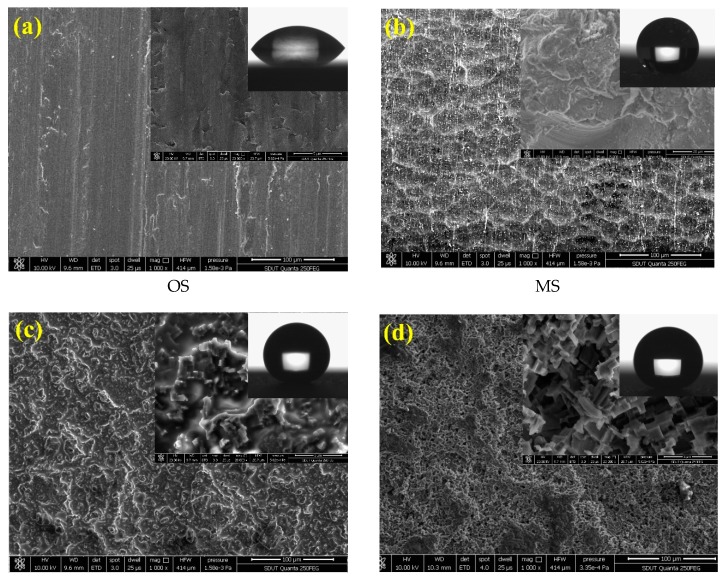
Surface morphologies of aluminum alloy samples. (**a**) Original microstructure before sand peening (SP) and electrochemical oxidation (ECO); (**b**) microscale pits structure generated by SP; (**c**) nanoscale corals structure formed by ECO; (**d**) micro-nanoscale binary structure fabricated by SP and ECO.

The combination of SP and ECO was carried out with the optimum parameters obtained from range analysis, i.e., 8 g/L of electrolyte concentration, 4 V of working voltage, 150 A/m^2^ of current density, and 3 h of reaction time, to investigate the growth process of the morphological structure on the sample surface, as shown in Figure 3. Meanwhile, the surface roughness Ra, water contact angle CA, and sliding angle SA of the as-prepared sample are listed in Table 4. Figure 3a shows the surface of the original Al alloy sample (OS). It can be seen that the bare surface was smooth, and there existed only a few textures scraped by metallographic sandpapers. Figure 3b shows the surface morphology of the primary sand-peened sample. The SP with 120 mesh of alumina particles and 5 mm of nozzle diameter at 0.65 MPa of impacting pressure for 150 s constructed several pits and protrusions with a size in the range of 50–100 μm. It can be observed that these pits and protrusions joined with each other, assembling into an interconnected concave–convex microstructure (MS). After the FAS modification, the water droplet formed a hemisphere on the as-prepared microstructure, as shown in Figure 3b. Figure 3c shows the morphological structure formed on the original surface following electrochemical oxidation with the electrolyte concentration of 6 g/L, working voltage of 4 V, and current density of 200 A/m^2^ for 2.5 h. It can be seen that the sample surface was covered with a great quantity of coral-like nanostructures, with sizes of tens to hundreds of nanometers (NS). The water droplet on the sample surface with NS became approximately spherical in shape. These coral-like nanostructures, which grew on micro pits, contributed to the formation of binary micro/nanoscale structures (BS), as shown in Figure 3d. Thus, the BS could be constructed successfully via a combination of SP and ECO. In Figure 3d, the surface roughness of BS was 1.32 μm, between that of MS (2.78 μm) and NS (0.41 μm). In addition, the surface morphology of BS was deemed to be beneficial for superhydrophobicity, due to the high amount of air trapped under the droplet.

Table 4 displays the CA of water droplets on the OS, MS, NS, and BS. It can be observed that the CA of OS was only 54.1° ± 3.3°, and the SA was more than 90°. After sand peening, the SA of MS went down to 13° ± 1.5°, but the CA of 148.4° ± 0.2° was less than 150°, which indicates that the wettability of MS switched from hydrophilic to hydrophobic. Although the water droplet on MS had the ability to roll off the surface, the MS had no superhydrophobic property. We can find that the CA of NS increased to 171.4° ± 0.5°, and the SA had a large value of 35° ± 1.3°. The results demonstrate that the NS had a higher CA, but the water droplet struggled to roll off the surface with NS. Compared with NS, BS was obtained via a combination of SP and ECO. The nanoscale structures obtained following ECO differed from the nanoscale structures fabricated directly via ECO (NS) on the sample without sand peening. In fact, after sand peening, some oxidation occurred on the surface of the aluminum alloy after shot peening, which hindered the electrochemical oxidation. This is potentially why the CA of BS was smaller than that of NS. Furthermore, from Figure 3 and Table 4, it can also be observed that the specific area and roughness of BS were greater than those of NS. In particular, the larger surface roughness would significantly reduce the influence of capillary force and negative pressure by nanoscale structures. This is potentially why the SA of BS was smaller than that of NS. Nevertheless, the CA of the water droplet on the BS reached 167.5° ± 1.1°, and the SA decreased to 2.5° ± 0.7°, due to the large amount of air trapped underneath the water droplet, induced by the combination of micro pits and coral-like nanostructure. From Table 4, it can be seen that the CA on the BS was obviously higher than 150°, and the SA was markedly lower than 10°. The results suggest that the as-prepared surface with BS had a significant self-cleaning property.

**Figure 4:**
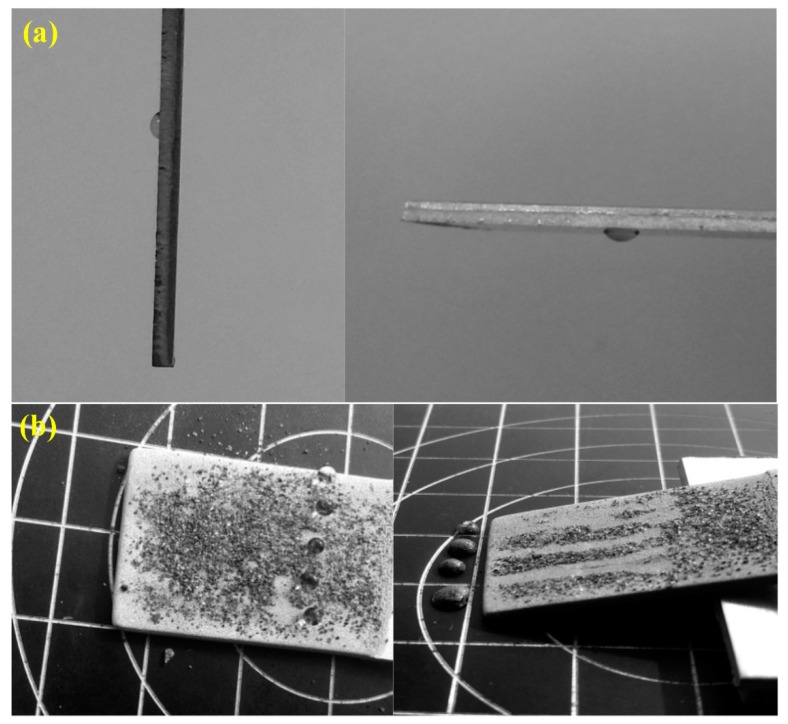
Self-cleaning performance of aluminum alloy samples with (**a**) OS and (**b**) BS obtained via a combination of SP, ECO, and fluoroalkylsilane (FAS) modification.

The test for the self-cleaning effect is illustrated in Figure 4. A water droplet was placed on the untreated sample surface, as shown in Figure 4a. Even if the sample was tilted at 90°, or even 180°, the water droplet could not roll down. In addition, brown alumina powders were evenly spread on the as-prepared sample with BS for the self-cleaning test, as shown in Figure 4b. It can be found that water droplets could easily roll down when the sample was inclined at an angle of 2.5° ± 0.7°. Then, the powders adhered to the water droplets and were carried away. This phenomenon could be investigated in detail using the Cassie–Baxter Model. The coral-like nanostructures produced via electrochemical oxidation had a high specific surface area, which led to a large amount of air being trapped. With the addition of pit-like microstructures generated by sand peening, water droplets could be almost suspended on the BS, indicating that micro/nanoscale binary structures markedly improved the self-cleaning performance.

### 3.3. Corrosion Resistance

**Figure 5:**
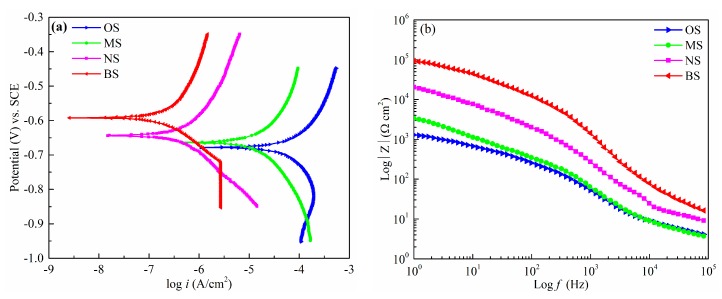
Electrochemical measurement of the OS, MS, NS, and BS in 3.5 wt.% NaCl solution. (**a**) Potentiodynamic polarization curves; (**b**) Bode plot (|Z| changes as a function of frequency ranging from 1 to 10^5^ Hz).

The potentiodynamic polarization curve is an effective method of characterizing the instantaneous corrosion rate of aluminum alloy samples. A higher corrosion potential denotes a lower corrosion current density and higher polarization resistance, corresponding to better corrosion resistance. Figure 5 shows the potentiodynamic polarization curves and Bode plots for sample surfaces with OS, MS, NS, and BS in 3.5 wt.% NaCl solution at room temperature. The corrosion potential and current density were calculated using Tafel curves, as listed in Table 5. 

Figure 5a shows the Tafel curves of OS, MS, NS, and BS. The corrosion potential of OS was −0.679 V, and the corrosion current density was 6.249 × 10^−4^ A/cm^2^. The corrosion potential of MS was 16 mV more positive than the OS, and the corrosion current density reduced to 1.071 × 10^−4^ A/cm^2^. The corrosion potential of NS was −0.634 V and it improved compared with the OS and MS, but it was worse than that of BS (−0.592 V). The corresponding current density decreased to 2.087 × 10^−6^ A/cm^2^, two orders of magnitude lower than OS and MS. From Figure 5a, it can be found that the corrosion potential and the current density (7.516 × 10^−7^ A/cm^2^) improved significantly when the sample surface became superhydrophobic after SP, ECO, and FAS modification. The corrosion potential of BS was 87 mV more positive than the OS and 71 mV higher than the hydrophobic surface with MS. Furthermore, the corrosion current density was decreased by three orders of magnitude as compared to that of OS. Figure 5b shows the Bode plots (amplitude–frequency) for OS, MS, NS, and BS. In the Bode plot, the amplitude–frequency curve of BS was significantly higher than the others. Obviously, the above data reveal that the as-prepared surface with BS had the lowest corrosion rate and the best corrosion inhibition. In addition, the inhibition efficiency could be calculated using Equation (1).

(1) $$\eta_{ie}=\frac{i_{corr}(OS)-i_{corr}(BS)}{i_{corr}(OS)}\times 100\%,$$

where, *i_corr_*(OS) and i*_corr_*(BS) are the corrosion current density of the sample surface with OS and BS. The inhibition efficiency of BS was 99.9% and higher than others. According to above results, the surface obtained via a combination of shot peening, electrochemical oxidation, and FAS modification showed superior anti-corrosion performance. The reason for the excellent corrosion resistance can be attributed to the binary structures and chemical compositions. The former was composed of hierarchical micro-nanostructures, and the water transport against gravity was easy in such a structure. The solution could be pushed out from the pores of the SHS via Laplace pressure, and the aluminum alloy substrate could be effectively protected [34]. It is well known that a surface fabricated via electrochemical oxidation is mainly composed of Al_2_O_3_ [35]. After the surface modification of FAS, some groups, such as –CF_2_, –CF_3_, and so on, were formed on the sample surface [36]. The film composed of aluminum oxides and low-energy groups can be regarded as a passivation layer, providing effective protection for aluminum alloys from being attacked by chloride ions. 

### 3.4. Chemical Stability

**Figure 6:**
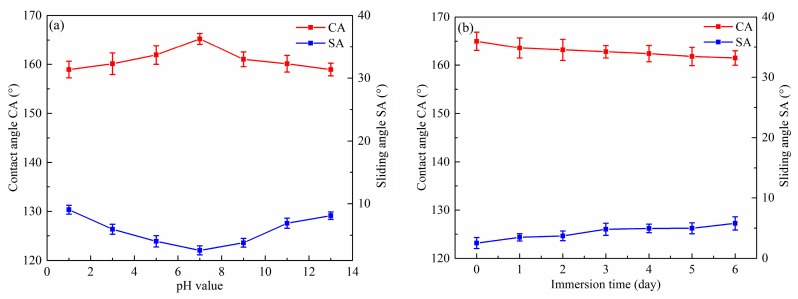
Influence of pH values (**a**) and immersion time in water (**b**) on the CA and SA of the as-prepared superhydrophobic surfaces with BS.

Superhydrophobic surfaces are always used in many alkaline and acidic environments. Figure 6a displays the CA and SA of the as-prepared surface as a function of the pH of the water droplet. The pH value of the water droplet was adjusted by changing the concentration of hydrochloric acid and sodium hydroxide. It is clear that the CA was higher than 150° and the SA was lower than 10°. Therefore, the as-prepared surface retained good superhydrophobicity in pH values ranging from 1 to 13. This suggests that the superhydrophobic surface had outstanding chemical stability in both acidic and alkaline environments. Figure 6b shows the change in CA and SA as a function of the immersion time in water. The CA slightly decreased to 160° from 167° and the SA marginally increased to 5.8° from 2.5° as the as-prepared surface was immersed in water for six days. It can be concluded that that the surface with BS was still superhydrophobic after being immersed in water for several days. 

## 4. Conclusions

A stable SHS was successfully fabricated on aluminum alloy via sand peening and electrochemical oxidation followed by low-energy modification. The wetting property varied with the process parameters of sand peening and electrochemical oxidation. The best CA and SA were 167.5° ± 1.1° and 2.5° ± 0.7°, respectively. The significant superhydrophobicity of the as-prepared surfaces was attributed to the synergistic effect of binary rough structures and the low-energy materials. A nanoscale coral-like structure was constructed via electrochemical oxidation. Combined with the microscale pit-like structure obtained via sand peening, it could hold water droplets on the as-prepared surface, achieving a transition from the Wenzel model to the Cassie–Baxter model. The as-prepared superhydrophobic surface has not only good self-cleaning performance and excellent corrosion resistance, but also outstanding chemical stability. Such a simple and feasible method could be easily applied to large-scale productions of aluminum alloy for industrial application.

## Figures and Tables

**Table 1:** Experimental conditions of shot peening.

Impact Pressure A (MPa)	Particle Size B (mesh)	Nozzle Diameter C (mm)	Impact Time D (s)
0.6	30	4	60
0.65	60	5	90
0.7	90	6	120
0.75	120	7	150

**Table 2:** Experimental conditions of electrochemical oxidation.

Working Voltage E (V)	Electrolyte Concentration F (g/L)	Current Density G (A/m^2^)	Oxidation Time H (h)
3	4	150	2
4	6	200	2.5
5	8	250	3

**Table 3:** Design of the orthogonal test and experimental results.

No.	A	B	C	D	CA (°)
1	0.6	30	4	60	138
2	0.6	60	5	90	135
3	0.6	90	6	120	139
4	0.6	120	7	150	142
5	0.65	30	6	150	139
6	0.65	60	7	120	140
7	0.65	90	4	90	141
8	0.65	120	5	60	148
9	0.7	30	7	90	140
10	0.7	60	6	60	138
11	0.7	90	5	150	143
12	0.7	120	4	120	139
13	0.75	30	5	120	143
14	0.75	60	4	150	139
15	0.75	90	7	60	138
16	0.75	120	6	90	140

**Table 4:** Surface roughness and wettability of the aluminum alloy sample with original sample (OS), microstructure (MS), nanostructure (NS), and binary structure (BS). Ra—roughness; SA—sliding angle.

Items	Original Al Alloy Sample (OS)	Microstructure (MS)	Nanostructure (NS)	Binary Micro/Nanoscale Structure (BS)
Ra (μm)	0.09	2.78	0.41	1.32
CA (°)	54.1 ± 3.3	148.4 ± 0.2	171.2 ± 0.5	167.5 ± 1.1
SA (°)	>90	13 ± 1.5	35 ± 1.3	2.5 ± 0.7

**Table 5:** Parameters of potentiodynamic polarization curves for OS, MS, NS, and BS.

Sample	*E_corr_* (V)	*i_corr_* (A/cm^2^)
OS	−0.679	6.249 × 10^−4^
MS	−0.663	1.071 × 10^−4^
NS	−0.634	2.087 × 10^−6^
BS	−0.592	7.516 × 10^−7^
